# PTRF Confers Melanoma‐Acquired Drug Resistance Through the Upregulation of EGFR

**DOI:** 10.1111/cpr.70086

**Published:** 2025-07-31

**Authors:** Miao Wang, Ying Cao, Chengcheng Ren, Ke Wang, Yaxiang Wang, Xiaoying Wu, Jian Mao, Qian Liang, Qian Zhang, Hezhe Lu, Xiaowei Xu, Yu‐Sheng Cong

**Affiliations:** ^1^ Zhejiang Key Laboratory of Medical Epigenetics, Zhejiang Key Laboratory of Aging and Cancer Biology Hangzhou Normal University School of Basic Medical Sciences Hangzhou China; ^2^ State Key Laboratory of Membrane Biology Institute of Zoology, Chinese Academy of Sciences Beijing China; ^3^ Department of Pathology and Laboratory Medicine, Perelman School of Medicine University of Pennsylvania Philadelphia Pennsylvania USA; ^4^ Hunan Provincial Key Laboratory of Basic and Clinical Pharmacological Research of Gastrointestinal Cancer The Second Affiliated Hospital, University of South China Hengyang China

**Keywords:** caveolae, drug resistance, EGFR, melanoma, PTRF

## Abstract

Melanoma is the most serious type of skin cancer. About half of all melanomas have activating BRAF mutations. Targeted therapy for malignant melanoma with BRAF inhibitor (BRAFi) or its combination with MEK inhibitor (MEKi) improves the clinical outcomes of patients, but resistance develops invariably. The underlying mechanisms remain incompletely understood. Here, we show that caveolae number is increased in both BRAFi and BRAFi + MEKi‐resistant melanoma cells, and the expression of the critical caveolae component PTRF is significantly upregulated in drug‐resistant melanoma cell lines and tumour tissues. Knockdown of PTRF in drug‐resistant cells reduces proliferation with increased apoptosis, whereas ectopic expression of PTRF confers resistance on parental cells to BRAFi or BRAFi + MEKi. On the contrary, the knockdown of PTRF in parental cells reduces their ability to acquire drug resistance induced by BRAFi treatment. Interestingly, we find that the expression of EGFR is increased along with PTRF and caveolin‐1 in drug‐resistant cells and in PTRF transduced parental cells, whereas knockdown of PTRF results in down‐regulation of EGFR expression and attenuates drug resistance of parental cells induced by PTRF expression. Together, these results suggest that PTRF contributes to therapy resistance through upregulating EGFR in melanoma cells.

## Graphical abstract

The upregulation of PTRF, a critical caveolar component, and the concomitant increase in caveolae number potentiate cellular proliferation and survival pathways, thereby contributing to melanoma drug resistance.

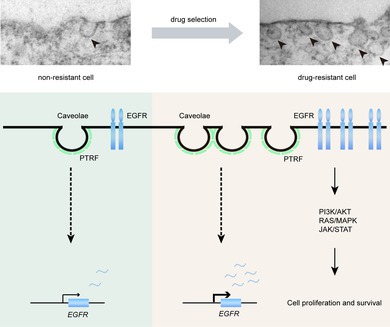

## 1 Introduction

Caveolae are 50–100 nm ‘flask‐shaped’ invaginations of the plasma membrane that play various physiological roles [1, 2, 3]. As a platform of signal transduction in the plasma membrane, caveolae are involved in a number of fundamental cellular processes, including senescence, tumorigenesis and lipid metabolism [4, 5]. Altered caveolae signalling has been linked to a wide range of human diseases, including lipodystrophy, muscular dystrophies, cardiac disease and cancer [3, 6]. Caveolin protein family members (caveolin‐1, 2, 3) are the primary structural components of caveolae. Caveolin‐1 is widely expressed and functions as a scaffolding protein in most cell types [3, 7]. The cavin protein family (cavin‐1, 2, 3, 4) is crucial to caveolae formation, stability and trafficking [8, 9]. Cavin‐1, originally named PTRF, acts as a peripheral component of caveolae that stabilises caveolin‐1. Expression of PTRF in PC3 cells, which express caveolin‐1 but lack PTRF and caveolae, is sufficient to induce the formation of caveolae [10]. Conversely, PTRF‐knockout mice exhibit global loss of caveolae and a lipodystrophic phenotype [11]. This suggests that PTRF is an indispensable prerequisite for caveolae formation and function.

Melanoma is the most serious type of skin cancer. About half of all melanomas have activating mutations in the BRAF oncogene, which is a component of the RAS/MAPK signalling pathway (RTK–RAS–RAF–MEK–ERK cascade) [12]. Targeted therapy for malignant melanoma with BRAF inhibitor (BRAFi) or its combination with MEK inhibitor (MEKi) improves the clinical outcomes of patients, but resistance develops invariably. Several mechanisms, including ERK reactivation and activation of pro‐survival or proliferation factors through PI3K/AKT and JAK/STAT pathways, have been reported to mediate drug resistance in cancer [13, 14, 15, 16, 17]. Activation of these pathways is often linked to the cell surface receptor tyrosine kinases such as EGFR. EGFR can be activated through autophosphorylation of its intracellular domain, leading to the activation of downstream signalling cascades including PI3K–AKT, MEK–ERK and JAK–STAT [18, 19]. Indeed, several reports suggest that the EGFR signalling pathway plays a critical role in melanoma drug resistance [20, 21, 22, 23]. However, the molecular mechanisms underlying melanoma‐acquired drug resistance remain incompletely understood.

Here, we show that PTRF expression and caveolae number are significantly increased in drug‐resistant melanoma cells. Our data suggest that PTRF confers melanoma‐acquired drug resistance through the upregulation of EGFR.

## 2 Results

### 2.1 Increase in PTRF Expression and Caveolae Number Is Associated With Melanoma‐Acquired Drug Resistance

**FIGURE 1:**
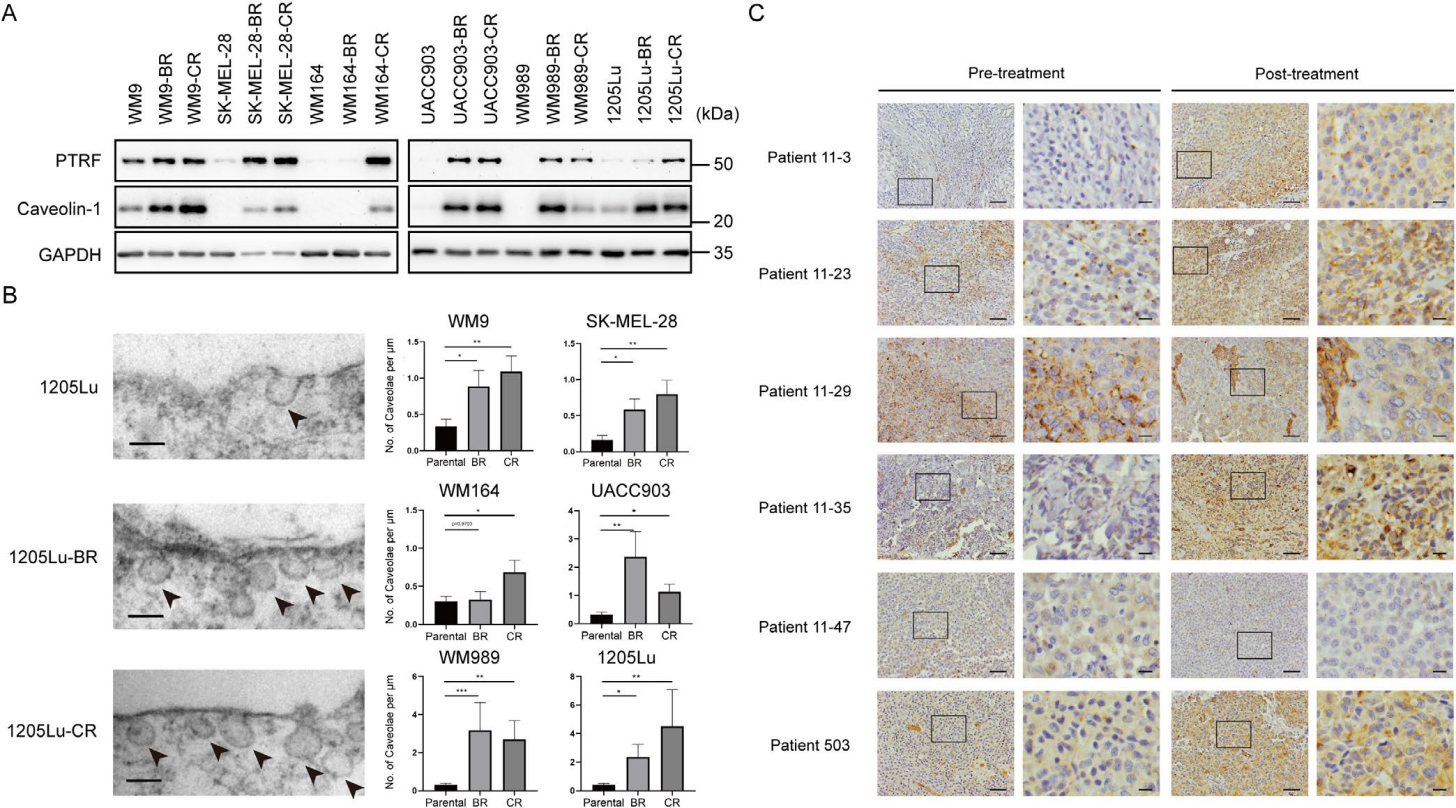
Increase in PTRF expression and number of caveolae are associated with melanoma‐acquired drug resistance. (A) Western blot analysis showing increased PTRF and caveolin‐1 expression in BRAFi‐resistant (BR) and BRAFi + MEKi‐resistant (CR) melanoma cells. (B) Drug‐resistant cells exhibited an increased number of caveolae. Left: transmission electron microscope (TEM) images of 1205Lu parental, BR and CR cells (scale bar represents 0.1 μm). Caveolae were indicated by arrows. Right: comparison of the number of caveolae in the indicated BR or CR cells versus the parental cells. The number of caveolae was statistically analysed in at least nine independent cells. Error bars represent SEM (*p < 0.05; **p < 0.01; ***p < 0.001; ns, no significance). (C) Immunohistochemical (IHC) analysis showing increased PTRF expression in paired tumour biopsies of the indicated patients before and after BRAFi treatment. Low‐magnification representative images are shown on the left (scale bar represents 100 μm) and higher magnification images are shown on the right (scale bar represents 20 μm) of pre‐ or post‐treatment tumour biopsy specimens.

Considering that caveolae are recognised as a cellular platform implicated in numerous signalling pathways, we examined the expression levels of the critical caveolae components PTRF and caveolin‐1 in melanoma cell lines resistant to BRAFi (BR) or to the combined therapy of BRAFi and MEKi (CR), and their respective parental cell lines. The results showed that both PTRF and caveolin‐1 were significantly upregulated in drug‐resistant cells compared with the parental cells (Figure 1A). Consistently, electron microscopic analysis showed an increased number of caveolae in drug‐resistant cells (Figure 1B). Immunohistochemistry staining analysis showed that PTRF expression was significantly increased in four out of six post‐treatment tumour biopsy specimens (Figure 1C). These results suggest that caveolae might be involved in the development of acquired drug resistance in melanoma.

### 2.2 PTRF Is Required for Proliferation and Survival of Drug‐Resistant Melanoma Cells

**FIGURE 2:**
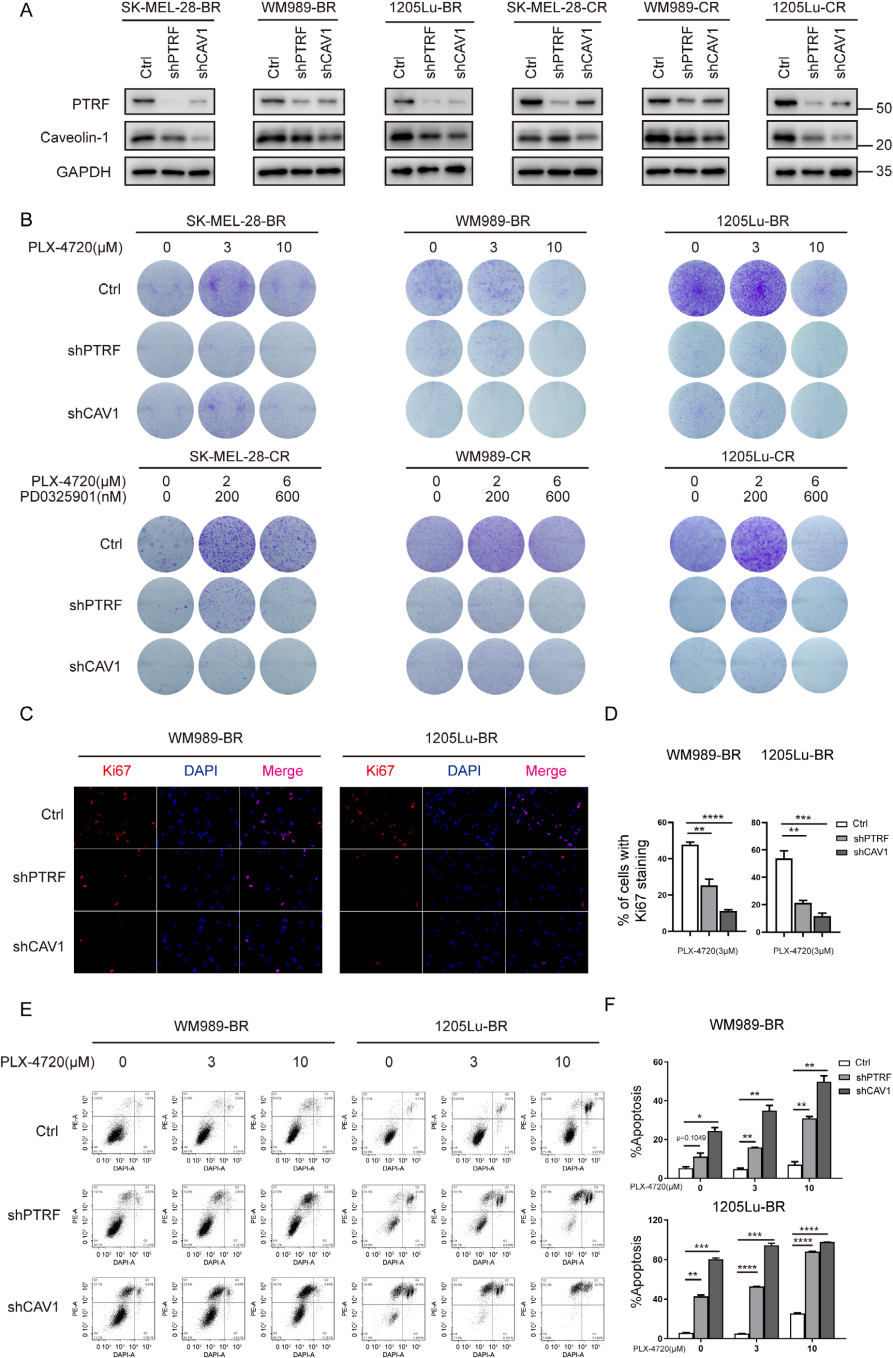
PTRF is required for proliferation and survival of drug‐resistant melanoma cells. (A) Western blot analysis of PTRF and caveolin‐1 levels in BR and CR cells targeted by the indicated shRNAs. (B) Knockdown PTRF or cavelin‐1 conferred a growth disadvantage to drug‐resistant cells. Cells were seeded in 6‐well plates and treated with drug(s) at indicated concentration for 8 days. The cells were fixed, stained with crystal violet and scanned. (C) Immunofluorescence (IF) staining of Ki‐67 in BR cells targeted by the indicated shRNAs. Cells were treated with 3‐μM PLX‐4720 for 72 h and immunostained with Ki‐67 antibody (red). The nuclei were stained with DAPI (blue). (D) Quantification of the cells with Ki‐67 staining in (C). The percentage of Ki‐67 positive cells was calculated from at least five randomly chosen fields. Data represent mean ± SEM of three biological replicates (*p < 0.05; **p < 0.01; ***p < 0.001; ****p < 0.0001). (E) FACS analysis of BR cells targeted by the indicated shRNAs. Cells were treated with PLX‐4720 at indicated concentration for 72 h, labelled with Annexin V‐PE/DAPI and analysed using BD LSRFortessa cell analysers. (F) Quantification of the apoptotic cells in (E). Data represent mean ± SEM of two biological replicates (*p < 0.05; **p < 0.01; ***p < 0.001; ****p < 0.0001).

**FIGURE 3:**
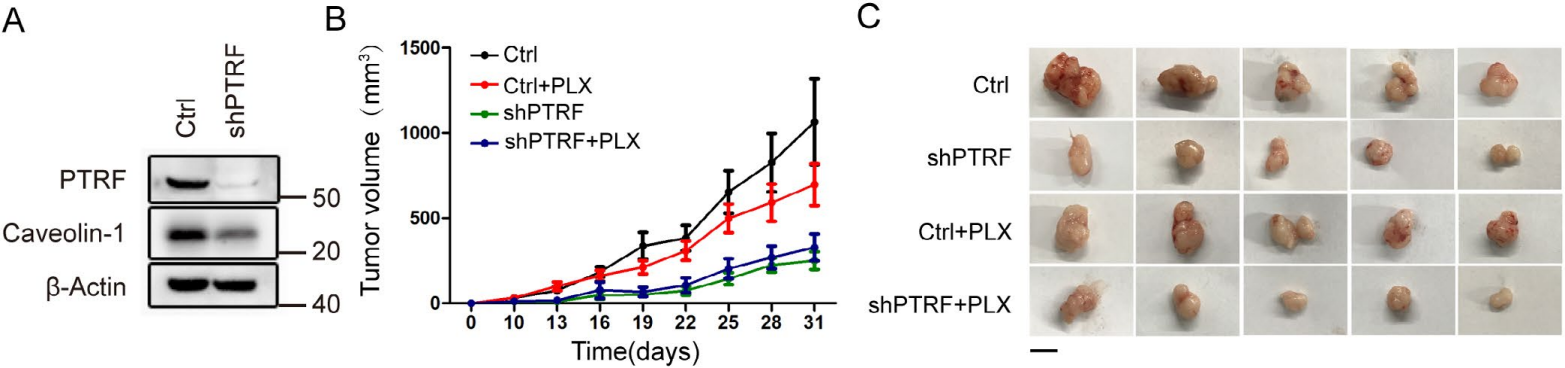
Depletion of PTRF reduced BRAFi resistant tumour xenograft growth. (A) Western blot analysis of PTRF and caveolin‐1 levels in 1205Lu‐BR cells transduced with shPTRF or control vectors. (B) Tumour growth curve of mice bearing xenografts. 1205Lu‐BR cells transduced with shPTRF or control vectors were injected into nude mice. After the mice had visible tumours within 10 days, both groups were randomly assigned to be administered PLX‐4720 or its corresponding control diet. Tumour growth was monitored and tumour volume was calculated every 3 days. (Ctrl: *n* = 10, Ctrl + PLX: *n* = 11, shP: *n* = 8, shP + PLX: *n* = 12). Two‐way ANOVA analysis demonstrated statistical significance: Ctrl versus shPTRF (*p* = 0.0024); Ctrl versus shPTRF + PLX (*p* = 0.0018) and Ctrl + PLX versus shPTRF + PLX (*p* = 0.0109). (C) Representative photographs of final dissected xenograft tumour masses (scale bar represents 1 cm).

Given that PTRF and caveolin‐1 are prerequisites for caveolae formation and function, we investigated the role of caveolae in melanoma drug resistance by knockdown of PTRF or caveolin‐1 in the drug‐resistant 1205Lu‐BR cell line. We found that the knockdown of PTRF or caveolin‐1 resulted in decreased colony formation ability with or without BRAFi PLX‐4720 treatment (Figure S1A–D), suggesting that caveolae are required for the proliferation of drug‐resistant melanoma cells. Similar results were found in several other BR and CR cell lines, in which the knockdown of either PTRF or caveolin‐1 inhibited cell proliferation and promoted apoptosis, as assayed by colony formation, Ki67 staining and fluorescence‐activated cell sorting (FACS) analysis (Figure 2). Conversely, the rescue experiment by ectopic expression of PTRF overcame the proliferation deficiency induced by PTRF knockdown in WM989‐BR and 1205Lu‐BR cell lines (Figure S1E–H). Furthermore, the knockdown of PTRF in drug‐resistant 1205Lu‐BR cells inhibited tumour growth in the mouse xenograft assay, with or without BRAFi treatment (Figure 3). Together, these results suggest that caveolae are essential for the proliferation and survival of acquired drug‐resistant melanoma cells.

### 2.3 PTRF Is Critical for the Acquisition of Melanoma Drug Resistance

**FIGURE 4:**
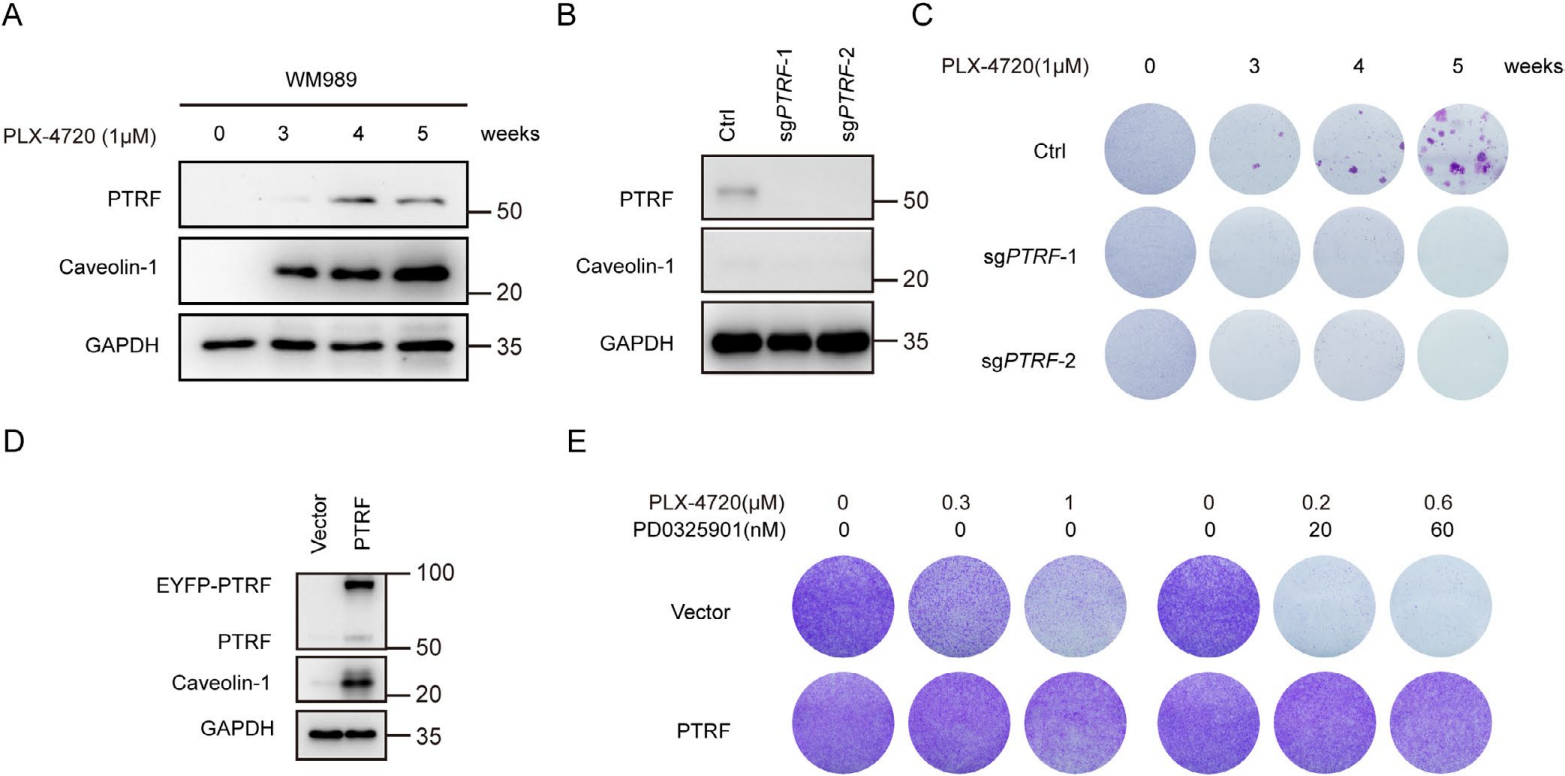
PTRF is required for the acquisition of drug resistance in primary melanoma cell lines. (A) Western blot analysis of PTRF and caveolin‐1 levels in WM989 cells during PLX‐4720 administration. (B, C) PTRF is necessary for WM989 parental cells to acquire resistance. Cells were transduced with two independent sgPTRF or control vectors. The levels of PTRF and caveolin‐1 were determined by Western blot analysis (B). Cells were seeded in 6‐well plates and treated with 1‐μM PLX‐4720 for 5 weeks. The cells were fixed, stained with crystal violet and scanned (C). (D, E) Expression of PTRF confers drug resistance to WM989 parental cells. Cells were transduced with EYFP‐PTRF or control vectors. The levels of PTRF and caveolin‐1 were determined by Western blot analysis (D). Cells were seeded in 12‐well plates and treated with drug(s) at the indicated concentration for 6 days. The cells were fixed, stained with crystal violet and scanned (E).

PTRF and caveolin‐1 are expressed at a low level in the parental melanoma cell line WM989, which is sensitive to BRAFi treatment. However, PTRF expression was increased following BRAFi PLX‐4720 treatment (Figure 4A). To investigate further the necessity of PTRF in the acquisition of drug resistance, we constructed PTRF depleted WM989–cell lines (Figure 4B). We chronically treated cells with PLX‐4720 to mimic the course of acquired drug resistance development. After 5 weeks of treatment, resistant colonies formed in the control group but barely formed in PTRF‐depleted cells (Figure 4C). On the contrary, ectopic expression of PTRF enabled WM989 to acquire drug resistance to BRAFi PLX‐4720 and to its combination with MEKi PD0325901 (Figure 4D,E). Hence, these results suggest that PTRF is critical for acquired drug resistance in melanoma.

### 2.4 PTRF Contributes to Melanoma‐Acquired Drug Resistance Through the EGFR Pathway

**FIGURE 5:**
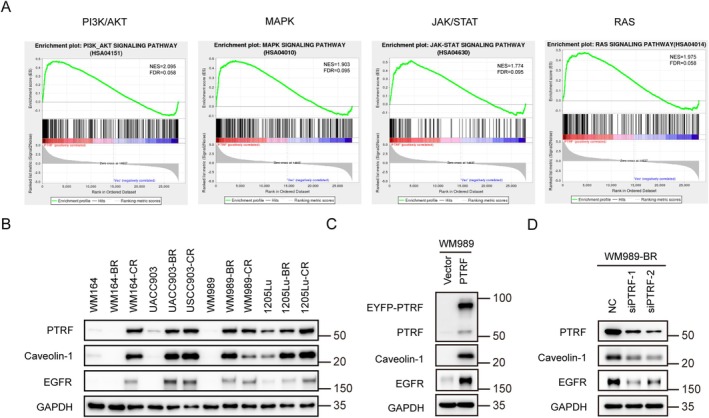
PTRF regulates EGFR pathway in melanoma cells. (A) Ectopic expression of PTRF activates PI3K/AKT, MAPK, JAK/STAT and RAS pathways in WM989 cells. Data were analysed using gene set enrichment analysis (GSEA) based on KEGG datasets. (B–D) Western blot analysis of PTRF, caveolin‐1 and EGFR protein levels in BR and CR melanoma cells (B), PTRF transduced WM989 cells (C) and PTRF‐knockdown WM989‐BR cells (D).

To explore the mechanism by which PTRF regulates drug resistance in melanoma cells, we performed RNA sequencing (RNA‐seq) analysis in the parental WM989 cells expressing exogenous PTRF. Compared with the control cells, four signalling pathways involved in cell proliferation and survival, namely PI3K/AKT, MAPK, JAK/STAT and RAS pathways, were significantly induced by PTRF upregulation (Figure 5A). Interestingly, these pathways are downstream of EGFR, which is frequently reported to be involved in melanoma drug resistance. We speculate caveolae may be involved in melanoma‐acquired drug resistance through EGFR pathway. To verify this connection, we examined the EGFR expression in parental, BR and CR cells. Compared to non‐resistant parental cells, EGFR was up‐regulated along with the increased PTRF and caveolin‐1 in resistant cells, both at the protein and RNA levels. (Figure 5B and Figure S2A,B). In addition, we observed that exogenous expression of PTRF in non‐resistant cells upregulated EGFR, whereas knocked down of PTRF in resistant cells downregulated EGFR (Figure 5C,D and Figure S2C,D).

**FIGURE 6:**
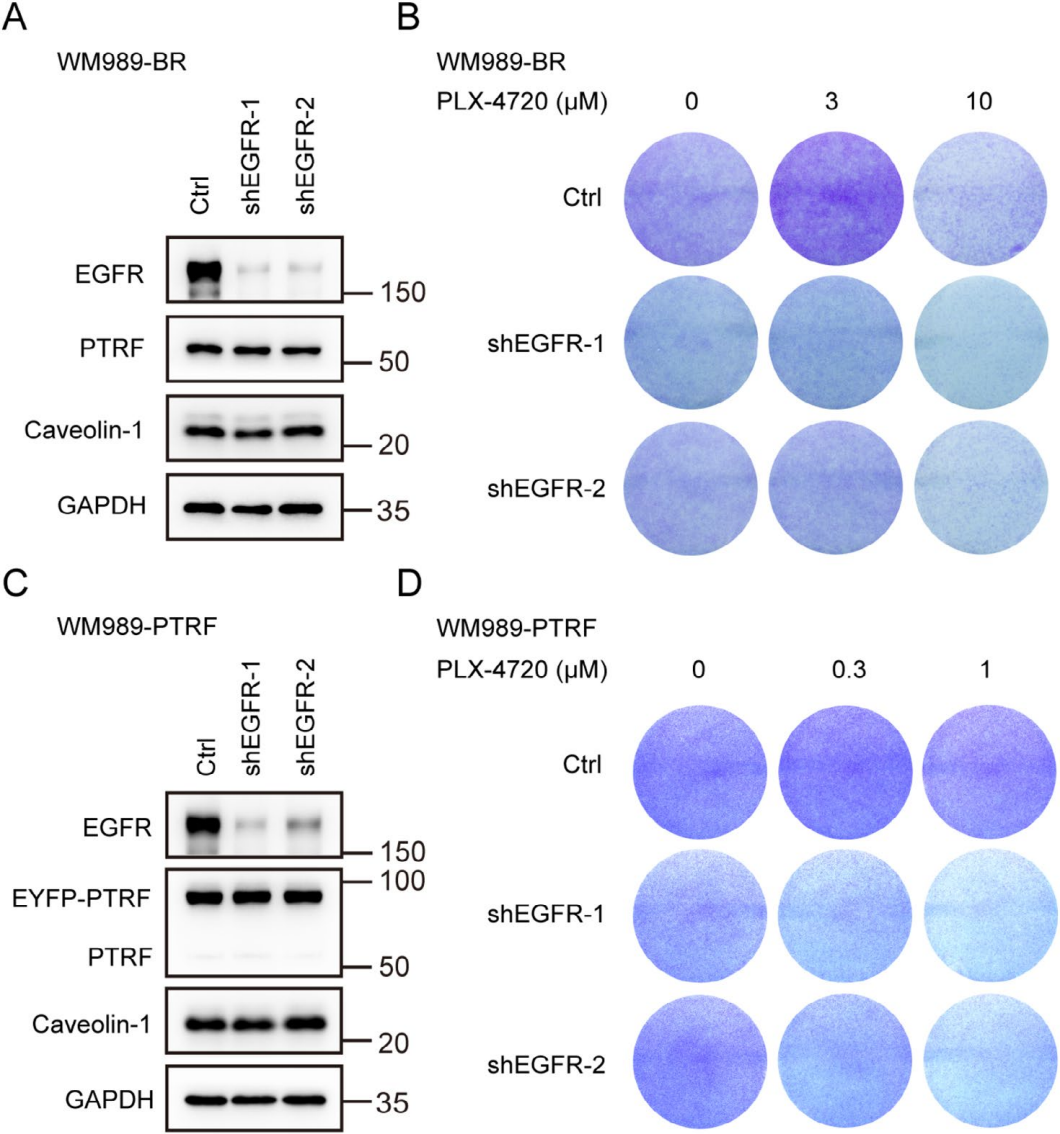
PTRF regulates drug resistance through EGFR. (A, B) WM989‐BR cells were transduced with the indicated shRNAs. EGFR, together with PTRF and caveolin‐1 protein levels, were analysed by Western blot (A). Cells were seeded in 6‐well plates and treated with indicated concentrations of PLX‐4720 for 10 days. The cells were fixed, stained with crystal violet and scanned (B). (C, D) WM989‐PTRF cells were transduced with the indicated shRNAs. Expression levels of EGFR, PTRF and caveolin‐1 were analysed by Western blot (C). Cells were seeded in 6‐well plates and treated with indicated concentrations of PLX‐4720 for 10 days. The cells were fixed, stained with crystal violet and scanned (D).

As ectopic expression of PTRF conferred a growth advantage to non‐resistant cells under drug treatment (Figure 4E), we attempted to investigate whether PTRF mediates drug resistance through EGFR by knocking down EGFR in resistant melanoma cells and found that knockdown of EGFR resulted in reduced drug resistance (Figure 6A,B). Furthermore, in cells ectopically expressing PTRF, which became resistant to BRAFi treatment (Figure 4E), knocking down EGFR expression restored sensitivity to drug treatment (Figure 6C,D). Together, these results suggest that PTRF induces acquired drug resistance in melanoma cells, at least in part, through EGFR up‐regulation.

## 3 Discussion

As a specialised membrane domain, caveolae modulate several signalling pathways through membrane receptors [5, 8, 24]. Among these receptors, EGFR has been frequently reported to be closely associated with melanoma drug resistance [20, 21, 22, 23]. Cells with high EGFR expression are more resistant to BRAFi [25, 26]. In this work, we found that caveolae number was increased, caveolar critical components PTRF and caveolin‐1 were upregulated in drug‐resistant melanoma cell lines. Immunohistochemistry staining analysis showed that PTRF expression was significantly increased in four out of six post‐treatment tumour biopsy specimens. However, due to the limited tumour sample size, the clinical significance of the correlation between PTRF expression and acquired drug resistance requires further investigation. Nonetheless, levels of PTRF expression were positively correlated with those of EGFR in acquired drug‐resistant melanoma cells. Ectopic expression of PTRF in drug‐sensitive cells conferred drug resistance, whereas knockdown of PTRF in drug‐resistant cells sensitised cells to BRAFi treatment. Furthermore, we showed that EGFR knockdown attenuated drug resistance in WM989 cells, which acquired resistance through the exogenous expression of PTRF. These results indicated that caveolae contribute to melanoma drug resistance, at least in part, through EGFR.

RNA‐seq results revealed that several pathways that promote cell proliferation and survival were up‐regulated by the expression of ectopic PTRF, including PI3K/AKT, MAPK, JAK/STAT and RAS pathways. Given that caveolae contain a variety of signal transduction molecules, including multiple components of these pathways [27, 28, 29], caveolae could interact directly with these components and thereby modulate the corresponding pathways and coordinately contribute to drug resistance.

It has been reported that PTRF enhanced temozolomide resistance by increasing the efflux of the drug through extracellular vesicles in glioblastoma [30], and PTRF was upregulated along with multidrug‐resistant (MDR) P‐glycoprotein, an ATP‐dependent drug efflux pump, in adriamycin‐resistant breast cancer MCF‐7/ADR [31]. Whether the accumulation of caveolae in the membrane could facilitate the efflux of drugs is worth investigating. Malignant melanoma in individuals over the age of 55 has a much poorer prognosis for melanomas of equal grade and stage than younger individuals [32]. Interestingly, we have reported previously that PTRF expression and caveolae number were increased in senescent cells [33]. It is possible that aged individuals with increased PTRF expression and caveolae number are more vulnerable to acquire targeted therapy resistance.

Caveolae as specialised plasma membrane invaginations that may regulate the EGFR through multiple mechanisms, such as compartmentalisation, endocytosis and trafficking and lipid environment influence. The detailed mechanisms by which caveolae contribute melanoma‐acquired drug resistance, besides the transcriptional regulation of EGFR, merit further investigation.

In summary, our study discloses a novel mechanism by which caveolae mediate melanoma‐acquired drug resistance potentially by modulating EGFR expression. These findings imply that caveolae could be a novel target in combination with current BRAFi therapy to improve the curative efficiency of melanoma.

## 4 Materials and Methods

### 4.1 Cell Culture

All cell lines were cultured in DMEM medium supplemented with 5% FBS and 1% penicillin/streptomycin. Resistant cells were maintained with PLX‐4720 (BRAFi) at 2 μM for BR cells or the combination of PLX‐4720 at 2 μM plus PD0325901 (MEKi) at 200 nM for CR cells throughout the experiments.

### 4.2 Constructs

Lentiviral shRNA constructs (psi‐LVRH1GH) of PTRF, caveolin‐1 and negative control were obtained from Genecopoeia. According to the product datasheets, the target sequences were as follows: shPTRF‐1, 5′‐aggtcagcgtcaacgtgaaga‐3′; shPTRF‐2, 5′‐gggacaagttgcgcaaatcct‐3′; shCAV1‐1, 5′‐gagcttcctgattgagattca‐3′ and shCAV1‐2, 5′‐gcaatgtccgcatcaacttgc‐3′. The plasmid of shPTRF‐3 in the rescue experiment (Figure S1E–H) was constructed by cloning shRNA sequence targeted the 3′‐untranslated region of PTRF mRNA into the psi‐LVRH1GH vector. The target sequence of shPTRF‐3 (GACACGACCAGGTTCTCAA) is not present in the PTRF expression construct. Thus, ectopic expression of PTRF should not be affected by the shRNA. Lentiviral sgRNA plasmids of PTRF were constructed using LentiCRISPR (pXPR_001) vector. The gRNA target sequences were as follows: sg*PTRF‐1*, 5′‐CGCTCGACAATATAGAGCGT‐3′; sg*PTRF‐2*, 5′‐AGAGCTGATCAAGTCGGACC‐3′.

### 4.3 Compounds and Antibodies

PLX‐4720 and PD0325901 were purchased from Selleck Chemicals and stock solutions were dissolved in dimethylsulfoxide. The following antibodies were used in this study. Caveolin‐1 and EGFR, from Cell Signalling Technology; GAPDH and β‐Actin, from HuaBio; Ki‐67, from Santa Cruz Biotechnology; PTRF, from Bethyl Laboratories (for Western blot) and Proteintech (for immunohistochemistry).

### 4.4 Tumour Samples

Samples were formalin‐fixed and paraffin‐embedded using standard protocols. Slides were subjected to antigen retrieval and immunohistochemically stained with an antibody against PTRF.

### 4.5 Transmission Electron Microscopy

Cells were gently scraped off culture dishes, fixed with 2.5% glutaraldehyde in PBS at 4°C overnight and post‐fixed with 1% osmic acid for 1 h. After dehydration with a graded series of ethanol, specimens were infiltrated and embedded using acetone and Spurr resin, sectioned, stained with uranyl acetate and alkaline lead citrate and observed in Hitachi Model H‐7650 TEM. Specimens for transmission electron microscopy observation were prepared and viewed in the Bio‐Ultrastructure Analysis Lab of the Analysis Center of Agrobiology and Environmental Sciences, Zhejiang University.

### 4.6 Crystal Violet Staining

Cells transduced by indicated constructs were plated in 6‐ or 12‐well plates, fixed with 4% formaldehyde and washed twice in PBS. After staining with crystal violet solution (Beyotime), cells were thoroughly washed in distilled water, air dried and scanned by HP scanjet G4010.

### 4.7 Immunofluorescence Staining

Cells were plated onto coverslips in 12‐well plates and fixed with 4% paraformaldehyde (PFA). The fixed cells were permeabilised with PBS containing 0.5% Triton X‐100, blocked with 3% BSA, and then incubated with primary antibodies diluted in PBS containing 3% BSA overnight at 4°C. After, cells were washed three times in PBS and incubated in fluorochrome‐conjugated secondary antibody diluted in 3% BSA for 1 h at room temperature in dark. Cells were stained with DAPI nuclear stains before mounting with mounting medium for fluorescence (Vector Laboratories).

### 4.8 Cell Apoptosis Assay

Cell apoptosis was analysed by FACS using PE Annexin V Apoptosis Detection Kit I (BD Pharmingen). Briefly, cells were treated with indicated concentrations of PLX‐4720 for 72 h. After trypsinisation, cells were washed twice with cold PBS and then resuspended in 1× binding buffer. Subsequently, cells were labelled with PE‐Annexin V and DAPI and analysed using a BD LSRFortessa flow cytometer.

### 4.9 Mouse Xenografts

shPTRF or control lentiviral vector‐transduced 1205Lu‐BR cells (5 × 10^6^ cells per mouse) were injected subcutaneously into the left posterior flanks of 6–8‐week‐old BALB/c nude mice. The mice had visible tumours within 10 days and were then assigned randomly to each treatment group: oral administration of PLX‐4720 (200 p.p.m.) or its corresponding control diet. Tumour formation was monitored every 3 days, and tumour volume based on calliper measurements was calculated using the following formula: tumour volume = length × width × width/2. All animal experiments were approved by the Animal Care and Ethics Committee at Hangzhou Normal University.

### 4.10 RNA Sequencing

Cells were transduced with EYFP‐PTRF or control vectors. Cell samples were collected and total RNA was isolated with TRIzol reagent (Invitrogen). The RNA‐seq was performed by Novogene. Briefly, mRNA was purified from total RNA using poly‐T oligo‐attached magnetic beads, followed by fragmentation, reverse transcription and cDNA synthesis. After adenylation of 3′ ends, cDNA fragments were ligated with adaptors, and 370–420‐bp fragments were isolated by AMPure XP system and amplified by PCR. Library quality was assessed on the Agilent Bioanalyzer 2100 system. The qualified libraries were sequenced on an Illumina Novaseq platform and 150‐bp paired‐end reads were generated.

### 4.11 Statistical Analysis

Statistical analyses were performed using Prism 8 (GraphPad Software). Two‐tailed Student's *t*‐test (parametric) or Mann–Whitney test (non‐parametric) were used to calculate the statistical significance unless otherwise indicated. Statistical details for specific experiments can be found in figure legends.

## Author Contributions

M.W. and Y.‐S.C. conceived and designed the experiments. M.W., Y.C. and C.R. performed the most experiments with help from K.W., Y.W., X.W., J.M., Q.L. and Q.Z. X.X. and H.Z. provided critical materials. M.W. and Y.‐S.C. wrote the manuscript.

## Disclosure

The authors have nothing to report.

## Conflicts of Interest

The authors declare no conflicts of interest.

## Supporting information


**Data S1.** cpr70086‐sup‐0001‐Supinfo.

## Data Availability

The data that support the findings of this study are available on request from the corresponding author. The data are not publicly available due to privacy or ethical restrictions.
