# Supplementary material for: PTRF Confers Melanoma‐Acquired Drug Resistance Through the Upregulation of EGFR
Source: Cell Prolif. 2025 Jul 31;59(2):e70086. doi: 10.1111/cpr.70086 (PMC12877943; doi:10.1111/cpr.70086)
Supplement: Supplementary file 1 — Data S1. cpr70086‐sup‐0001‐Supinfo. [file CPR-59-e70086-s001.docx]

**Supplementary Materials**:

PTRF confers melanoma-acquired drug resistance through the upregulation of EGFR

Miao Wang^1^, Ying Cao^1^, Chengcheng Ren^1^, Ke Wang^1^, Yaxiang Wang, Xiaoying Wu^1^, Jian Mao^1^, Qian Liang^1^, Qian Zhang^1^, Hezhe Lu^2^*, Xiaowei Xu^3^*, Yu-Sheng Cong^1,4^*

^1^Zhejiang Key laboratory of Medical Epigenetics, School of Basic Medical Sciences, Hangzhou Normal University, Hangzhou, China

^2^State Key Laboratory of Membrane Biology, Institute of Zoology, Chinese Academy of Sciences, Beijing, China

^3^Department of Pathology and Laboratory Medicine, Perelman School of Medicine, University of Pennsylvania, Philadelphia, Pennsylvania, USA

^4^Hunan Provincial Key Laboratory of Basic and Clinical Pharmacological Research of Gastrointestinal Cancer, the Second Affiliated Hospital, University of South China, Hengyang, Hunan, China.

*For correspondence: Hezhe Lu (Hezhe@ioz.ac.cn), Xiaowei Xu (xug@mail.med.upenn.edu), Yu-Sheng Cong (yscong@hznu.edu.cn),

**Supplementary Figures**

Figure S1 related to Figure 2

Figure S2 related to Figure 5

**Figure S1**


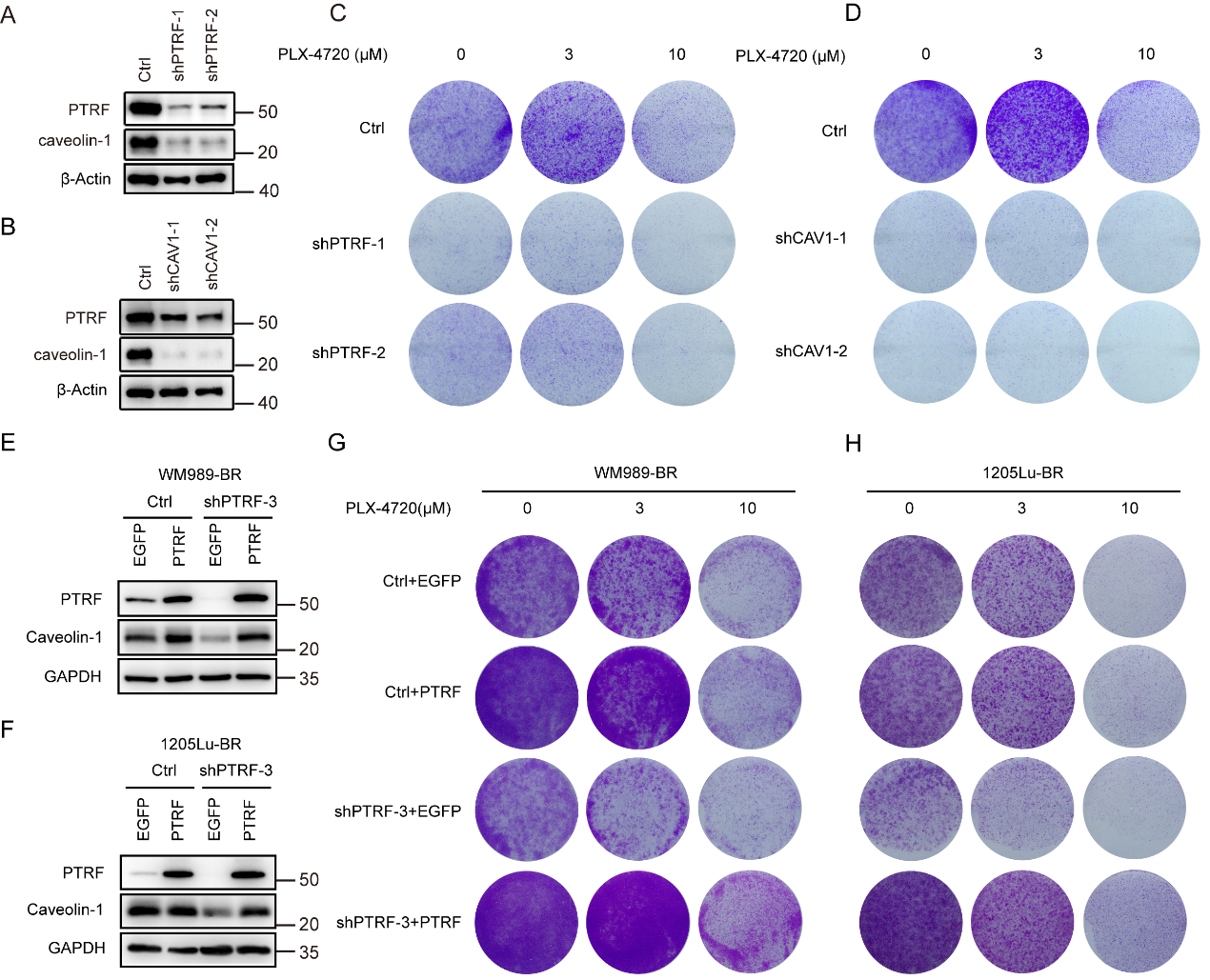


**Figure S1. PTRF confers a growth advantage to drug-resistant melanoma cells.** (A-D) Effects of PTRF or caveolin-1 suppression in BR cells. shRNAs targeting PTRF or caveolin-1 conferred a growth disadvantage to 1205Lu-BR cells. (A and B) Western blot analysis of PTRF or caveolin-1 levels in 1205Lu-BR cells targeted by the indicated shRNAs. (C and D) Cells were seeded in 6-well plates and treated with indicated concentrations of PLX-4720 (BRAFi) for 8 days. The cells were fixed, stained with crystal violet and scanned. (E-H) Ectopic expression of PTRF reversed the PTRF suppression-mediated growth disadvantage in BR cells. WM989-BR or 1205Lu-BR cells were transduced with a control lentiviral vector (EGFP) or a vector expressing PTRF following shRNAs transduction. (E and F) The levels of PTRF and caveolin-1 were determined by western blot analysis. (G and H) Cells were seeded in 6-well plates and treated with PLX-4720 at indicated concentration for 2 weeks. The cells were fixed, stained with crystal violet and scanned. The plasmid of shPTRF-3 in (E-H) is described in Materials and methods

**Figure S2**


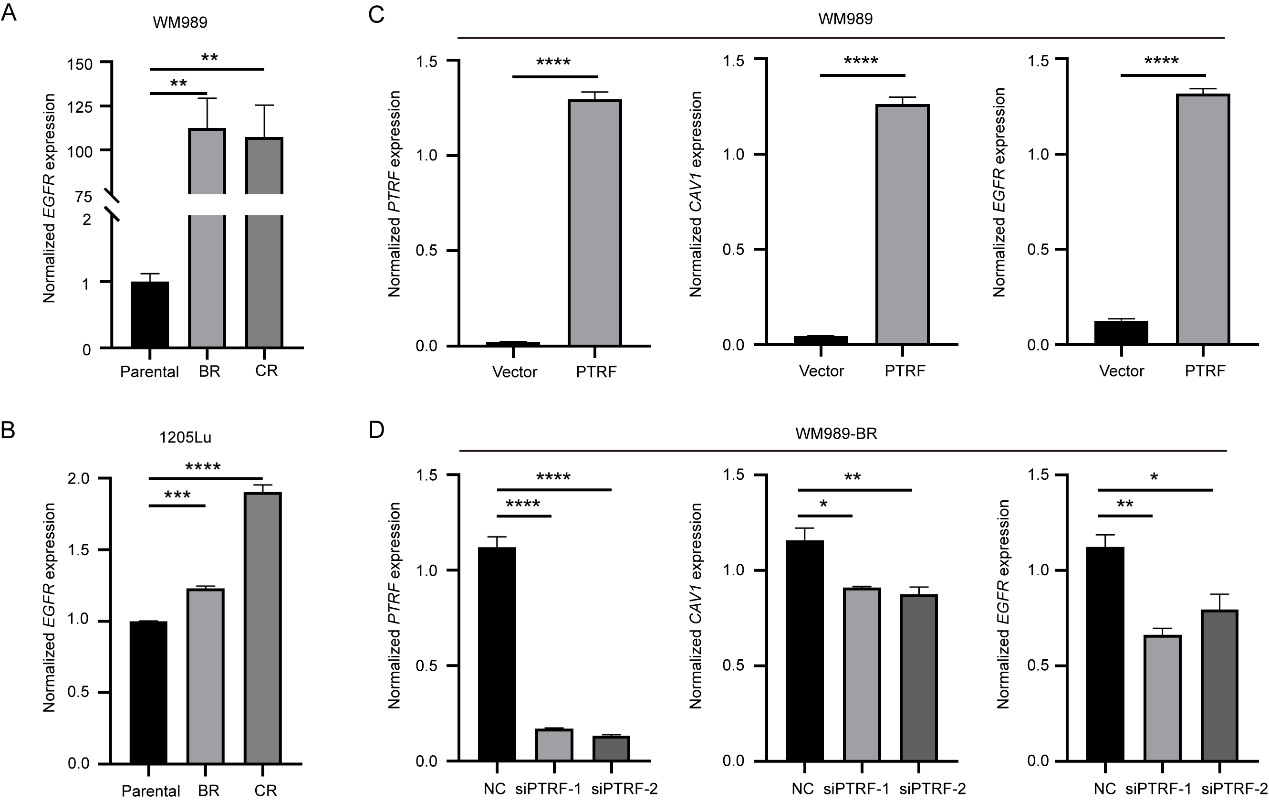


**Figure S2. PTRF regulates EGFR expression at the transcriptional level.** Realtime PCR analysis of EGFR levels in WM989 and 1205Lu parental, BR and CR cell lines (A and B), PTRF transduced WM989 cells (C) and PTRF-knockdown WM989-BR cells (D). For statistical analysis, two-tailed Student’s *t*-test (A-C) and one-way ANOVA (D) were used. Data represent mean±SEM of three biological replicates. (*p<0.05; **p <0.01; ***p<0.001; ****p<0.0001).
